# Antioxidant and Anti-Inflammatory Activities of Fractions from *Bidens engleri* O.E. Schulz (Asteraceae) and *Boerhavia erecta* L. (Nyctaginaceae)

**DOI:** 10.3390/medicines5020053

**Published:** 2018-06-12

**Authors:** Moussa COMPAORE, Sahabi BAKASSO, Roland Nâg Tiero MEDA, Odile Germaine NACOULMA

**Affiliations:** 1 Laboratory of Applied Biochemistry and Chemistry, University Ouaga I JKZ, Ouagadougou 03 BP 7021, Burkina Faso; odilenacoulma@yahoo.com; 2 Culture Platform of Cell and Tissue (PCCT) U.F.R/S.V.T., University Ouaga I JKZ, Ouagadougou 09 BP 1001, Burkina Faso; 3 Laboratory of Natural Products and Organic Synthesis, Department of Chemistry, Faculty of Science and Technology Abdou Moumouni University of Niamey, Niamey BP 10662, Niger; b_sahabi2000@yahoo.fr; 4 Laboratory for Research and Education in Animal Health and Biotechnology, University Nazi Boni of Bobo-Dioulasso, Bobo-Dioulasso 01 BP 1091, Burkina Faso; meda_roland@yahoo.fr

**Keywords:** antioxidants, cyclooxygenase, lipoxygenase, phenolic, flavonoid, traditional medicine

## Abstract

**Background:** According to recent studies, reactive oxygen is the leader of human metabolic disease development. The use of natural antioxidants is the best way to stop or prevent this problem. Therefore, the aim of this study was to evaluate the antioxidant and anti-inflammatory activities and to determine the polyphenolic contents of the *Bidens engleri* and *Boerhavia erecta* fractions. **Methods:** Plant fractions were obtained using Soxhlet procedures with hexane, dichloromethane, acetonitrile, ethyl acetate, methanol, and butanol solvent, successively. The different fractions were compared according to their antioxidant, anti-inflammatory activities, total phenolic, and total flavonoid contents. The phenolic contribution to the biological activity was evaluated. **Result:** The *Bidens engleri* and *Boerhavia erecta* fractions showed the highest antioxidant abilities, notably the polar fractions, which inhibited significantly the radical 2,2-diphenyl-1-picrylhydrazyl (DPPH) and 2,2-*O*-azinobis(3-ethylbenzoline-6-sulphonate) (ABTS). The butanol fraction from *Bidens engleri* and methanol fraction from *Boerhavia erecta* have presented the best iron (III) reduction power with 211.68 and 198.55 mgAAE/g, respectively. Butanol and acetonitrile were the best solvents for extracting phenolic compounds from *Bidens engleri* and *Boerhavia erecta*, respectively. In contrast, dichloromethane was the best solvent for extracting a flavonoid from two plants with anti-COX-2 and anti-LOX-15 active compounds. The phenolic compound contributed significantly to antioxidant activity (r > 0.80). **Conclusion:** The *Bidens engleri* and *Boerhavia erecta* fractions possessed a potential antioxidant for fighting oxidative stress and helping to prevent diabetes, hypertension, and cardiovascular diseases. The uses of this plant could be promoted in Burkina Faso.

## 1. Introduction

Oxidative stress is an inevitable consequence of life in an oxygen-rich atmosphere. The environment is filled with a lot of reactive oxygen species (ROS) and reactive nitrogen species (RNS). In recent data, it was demonstrated that ROS and RNS played an important role in human disease development [1]. In Burkina Faso, some recent studies have shown some alarming data concerning the prevalence of metabolic diseases [2]. The oxidative stress plays a direct or indirect role in the pathophysiology of diseases, such as cancer, diabetes, and cardiovascular diseases [3,4]. However, the intake of natural antioxidants has been reported to reduce the risk of cancer, cardiovascular diseases, diabetes, and other diseases that are associated with aging [5,6].

Plants, fruits, vegetables, and medicinal herbs possess a wide variety of free radical scavenging biomolecules, such as phenolic compounds, flavonoids, vitamins, terpenoids, and some other endogenous phytometabolites, which are rich in antioxidant capacity [7,8,9]. *Bidens engleri* and *Boerhavia erecta* were some well-known medicinal plants in the Central Plateau, because they were used in the treatment of diabetes mellitus, hypertension, and old wounds [10]. According to Nacoulma’s investigations, the traditional healers and herbalists used *Bidens engleri* and *Boerhavia erecta* in combination, for treating diabetes in Burkina Faso [10]. The same information was found in Cote d’Ivoire by ethnobotanical investigations [11].

The previous data demonstrated the antioxidant and anti-diabetic activities of *Boerhavia erecta* in India were associated with some polyphenolic compounds, such as phenolics and flavonoids [12,13]. Some antioxidant compounds, such as (+)-catechin (−)-epicatechin, quercetin, isorhamnetin, rutin, narcissin, isoquercitrin, and isorhamnetin 3-*O*-*β*-d-glucopyranoside, as well as other metabolites, were isolated from *B. erecta* leaves extract [14,15]. According to the medicinal importance of *B. engleri* and *B. erecta* in Burkina Faso, the present study aimed to highlight the potential of this plant by determining the antioxidant and anti-inflammatory activities, and the polyphenolic content of six organic fractions for identifying the type of metabolites that were responsible for biological activity.

## 2. Materials and Methods

### 2.1. Plant Material

Whole plants of *Bidens engleri* and *Boerhavia erecta* were taken from the Gampela region, which was situated in the mid-east of Kadiogo (central region), during the rainy season (August–September 2012). The sample was dried in the laboratory under ventilation. The sample was certified by Professor Jeanne MILLOGO, a botanist from the Laboratory of Plant Biology and Ecology (University of Ouagadougou). The herbaria were saved in the University Herbarium with numbers MC_501 and MC_502 for *Bidens engleri* and *Boerhavia erecta*, respectively.

### 2.2. Reagents and Solvents

The Folin–Ciocalteu reagent, sodium phosphate mono- and di-basics, sodium tetraborate, potassium persulfate, aluminum trichloride, trolox, 2,2-diphenyl-1-picrylhydrazyl (DPPH), 2,2-*O*-azinobis(3-ethylbenzoline-6-sulphonate) (ABTS), gallic acid, and trichloro acetic acid (TCA) were purchased from Sigma-Aldrich (Berlin, Germany). The sodium carbonate, potassium hexacyanoferrate, ascorbic acid, and ferric chloride were from Prolabo (Paris, France). The colorimetric COX (ovine) inhibitor screening assay kit, 15-lipoxygenase (soybean P1), linoleic, and arachidonic acids were purchased from Sigma-Aldrich, (New York, NY, USA).

### 2.3. Extraction Procedures

Of the sample, 20 g were successively extracted using hexane, dichloromethane, acetonitrile, ethyl acetate, methanol, and butanol in a Soxhlet system. The solvent was removed in a rotary evaporator system.

### 2.4. Antioxidant Effects Evaluation

#### 2.4.1. Radical DPPH Inhibition Determination

The fractions’ capacities to inhibit radical DPPH were evaluated according to the method that was presented by Compaoré et al. [16]. In a 96 micro-well plate, 200 µL of DPPH (20 mg/L) and 100 µL of fraction were incubated in the dark for 10 min, and the absorbencies were read at 517 nm using a spectrophotometer (BioTek Instruments, New York, NY, USA). Quercetin was used to generate a standard curve (*y* = −27.94 + 8.15, *r*^2^ = 0.99, *p* < 0.0001). The results were expressed in milligram Quercetin equivalent per gram (mgQE/g).

#### 2.4.2. Trolox Equivalent Antioxidant Capacity Assay

The method that was described by Compaoré et al. was used to evaluate the sample scavenging ABTS ability [16]. To 200 μL of diluted ABTS solution, 50 μL of fraction or trolox was added, with incubation in the dark for 5 min. The absorbance was read at 734 nm, with a microplate reader (BioTek Instruments, New York, NY, USA). Trolox was used to generate the standard curve (*y* = −72.38*x* + 54.57, *r*^2^ = 0.99, *p* < 0.001) and the results were expressed in millimole Trolox equivalent per gram (mMTE/g).

#### 2.4.3. Ferric (Fe III) Reducing Antioxidant Power (FRAP) Assay

The reducing power of the extracts was determined according to the method that was presented by Compaoré et al. [16]. The data were transformed to mg of ascorbic acid per gram of fraction (mgAAE/g), because the standard curve was obtained with ascorbic acid (*y* = 105.9*x*, *r*^2^ = 0.99, *p* < 0.0001). The iron (III) reducing activity of each sample was obtained from two of the three independent determinations.

### 2.5. Anti-Inflammatory Tests

#### 2.5.1. COX-1 and COX-2 Inhibition Assay

The inhibition of COXs was performed using a commercially available colorimetric COX (ovine) inhibitor screening assay kit (Cayman Chemical Company, New York, NY, USA). All of the inhibitors were dissolved in an appropriate solvent. The COX activity was evaluated using *N*,*N*,*N*’*N*’-tetramethyl-*p*-phenylenediamine (TMPD) as a co-substrate, with arachidonic acid. The TMPD oxidation was monitored spectrophotometrically at 590 nm (BioTek Instruments, New York, NY, USA). The inhibition percentage that was induced by 100 μg/mL of the sample was calculated.

#### 2.5.2. Lipoxygenase 15 Inhibition Assay

The assay was performed according to the previous procedure that was presented by Compaoré et al. [17]. The incubation mixture consisted of the sample solution (100 μg/mL) in an appropriate solvent and 200 μL of the enzyme solution (167 U/mL) in a boric acid buffer (0.2 M, pH 9). After the incubation at room temperature for 5 min, the reaction was started by adding 250 μL of linoleic acid solution (250 mM in buffer). The conversion of linoleic acid to 13-hydroperoxylinoleic acid was recorded by measuring the samples’ absorbencies at 234 nm, during 3 min, and against the appropriate blank solutions, without extracts. The inhibition percentage was calculated.

### 2.6. Polyphenolic Amount Quantification

#### 2.6.1. Phenolic Content Determination

The total phenolic content was evaluated using a Folin–Ciocalteu colometric assay, as described by Compaoré et al. [16]. The sample was mixed with Folin-Ciocalteu Reagent (0.2N). After incubation in the dark, 100 µL of sodium carbonate was added. The absorbance (760 nm) was measured after a second incubation in the dark (2 h), using the Biotek equipment (BioTek Instruments, New York, NY, USA). Gallic acid was used to produce the standard curve (*y* = 201*x* − 21.22, *r*^2^ = 0.99, *p* < 0.0001) and the results were expressed in mg gallic acid, equivalent per gram (mgGAE/g) of extract.

#### 2.6.2. Total Flavonoid Content Evaluation

The total flavonoid content was determined according to the previous method that was described by Compaoré et al. [16]. Then, 100 μm of sample and 100 μL of AlCl_3_ (2%) were mixed in 96 micro-wells and were incubated for 10 min. The absorbance was measured at 415 nm with a microplate reader (BioTek Instruments, New York, NY, USA). Quercetin was used to generate the standard curve (*y* = 39.8*x* − 3.5, r^2^ = 0.99, *p* < 0.0001) and the results were expressed at mg quercetin equivalent per gram (mgQE/g) of sample.

### 2.7. Statistical Analyses

Microsoft Excel was used to calculate the average and standard deviation of the repeated tests (*n* = 2 × 3). GraphPad Prism 6.01 (San Diego, CA, USA, 2012) and Xlstat Pro 7.5 (Paris, France, 2005) were used to produce the standard curve and to measure the statistical significant results, respectively (*p* < 5%).

## 3. Results and Discussion

### 3.1. Antioxidant Activities

The use of medicinal plants in Burkina Faso has been a current activity of the population [18]. However, the main role of the researchers was to promote the medicinal uses. The plant extracts’ antioxidant potential is shown in Table 1. The radical DPPH scavenging effect was decreased from 63.94 mgQE/g to 2.25 mgQE/g, and the radical ABTS scavenging power was decreased from 22.86 mMTE/g to 7.16 mMTE/g. The hexane fractions presented radical ABTS scavenging activities contrary to the anti-DPPH radical effect. The butanol fraction from *Bidens engleri* demonstrated the best antiradical possibility, similar to the acetonitrile from *Boerhavia erecta*. The ability of the fractions to reduce iron (III) were increased from 10.20 mgAAE/g to 211.68 mgAAE/g. In general, the *B. erecta* sample presented some antioxidant activity that was superior to the *B. engleri* samples. This data demonstrated the importance of these plant samples in stress oxidative management. In the previous data, it was demonstrated that *B. erecta* possessed some antioxidant activity that was supported by the flavonoid compounds [14,19]. The anti-DPPH, anti-ABTS, and iron (III) reduction abilities were evaluated [13,20]. However, it was the first antioxidant activity data from *Bidens engleri*, according to our bibliographic survey. According to previous antioxidant activities of similar fractions from *Commifora africana* (A. Rich.) Engl. (Burseraceae) and *Loeseneriella africana* (Willd.) (Celastraceae), which were from the same region, the present plants possessed a lowest antioxidant power [16].

### 3.2. Anti-Inflammatory Activity

Table 2 presents the data concerning the inhibition of prostaglandin production from COXs and LOX-15 regular activities. COX-2 was more sensitive than COX-1 and LOX-15, which were not sensitive to the *B. erecta* fractions. The percentage inhibition of COX-2 was from 23.65% to 64.72% at 100 µg/mL, as the final concentration of the fraction. The LOX-15 inhibition percentage was increased from 36.76 to 64.90%. The maximal inhibition of COX-1 was obtained with ethyl acetate (42.51%) from *B. engleri*. Interestingly, the dichloromethane fractions from *B. engleri* and *B. erecta* were the active fractions for COX-2 (64.72 ± 2.13%) and LOX-15 (62.55 ± 5.09%), respectively, according to the enzyme activity classification scale [21]. According to the previous data, *B. erecta* possessed some anti-inflammatory activity in vivo [22], but in the present study, the *B. erecta* fractions could not significantly inhibit the prostaglandin production from the COXs and LOX-15 activity. It was suggested that the enzyme inhibition was not the method of action of this anti-inflammatory effect. This was the first study of the evaluation of the COXs and LOX-15 inhibition power of two plants. These enzyme inhibition activities of the *B. engleri* fractions were very little compared with the *Commifora africana* and *Loeseneriella africana* fractions inhibition effect [16]. In contrast, the butanol and dichloromethane fractions from *B. engleri* presented some interesting inhibition activity of LOX-15, compared with the *Bauhinia rufescens* extract, Lam. (Caesalpiniaceae) [17].

### 3.3. Total Phenolic and Total Flavonoid Contents

**Figure 1:**
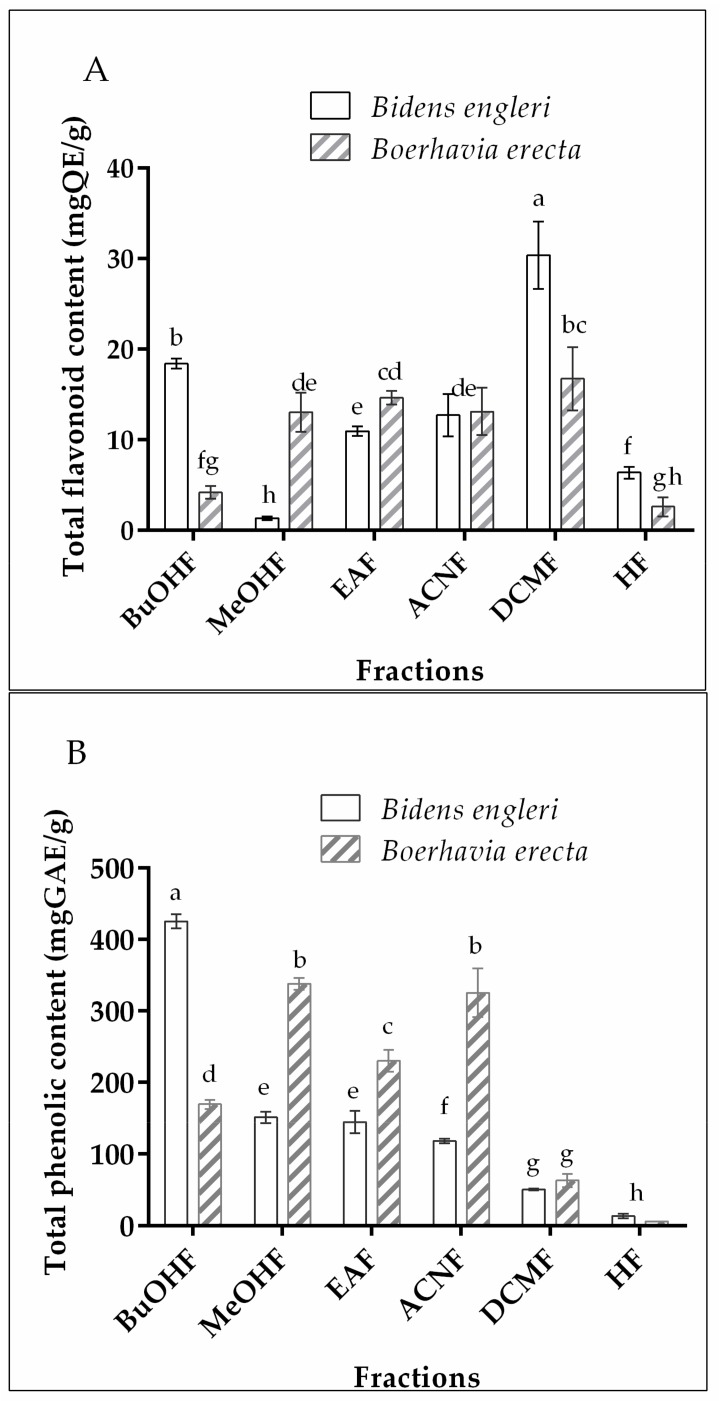
Polyphenolic content of fractions. A—total flavonoid contents; B—total phenolic contents. The data were obtained in two independent triplate tests (*n* = 2 × 3). The data in each histogram were statistically different (*p* < 0.05), except for the data with the same letters (a–h). BuOHF: butanol Fraction, MeOHF: methanol fraction, EAF: ethyl acetate fraction, ACNF: acetonitrile fraction, DCMF: dichloromethane fraction, HF: hexane fraction, mgQE/g: milligram quercetin equivalent per gram, mgGAE milligram gallic acid equivalent per gram.

As the metabolites were the main contributor to the antioxidant and anti-inflammatory powers, the phenolic and flavonoid contents were evaluated [16,23]. The yield of extraction is shown in Table 1. The methanol was the best solvent for extracting some metabolites from two plants, with a yield that was superior to 100 mg/g. Figure 1 shows the amount of flavonoid and phenolic in all of the fractions from *B. erecta* and *B. engleri*. The phenolic content was decreased from 425.12 to 5.92 mgGAE/g, and the flavonoid amount was increased from 2.62 to 30.38 mgQE/g. A notable variable distribution of polyphenolic compounds was found in concordance with the solvent polarities. Notably, *B. engleri* contained some non-polar flavonoids in the major compound that were extracted in dichloromethane, in contrast to *B. erecta*, which presented some polar compounds that were extractible by acetonitrile, ethyl acetate, and methanol. In previous phytochemical investigations, the flavonoid and phenolic contents were evaluated in the extracts from *B. erecta*. It was found that the ethanol and phosphate buffer were able to extract the flavonoid and phenolic compounds [13,14]. The flavonoid and phenolic individual compounds, with a radical scavenging activity and iron (III) reduction ability, were previously detected in the *B. erecta* extracts [24,25,26]. It was quercetin and isorhamnetin and their glycosides, rutin, narcissin, isoquercitrin, and isorhamnetin 3-*O*-*β*-d-glucopyranoside, as well as the two flavan-3-ols, [(+)-catechin] and [(−)-epicatechin], that are well known antioxidant phytometabolites [24,25,26]. These compounds showed anti-COX and anti-LOX properties [27,28].

The correlation analysis showed that phenolic contributed significantly to the radical scavenging and iron (III) reduction. The contribution to the anti-DPPH, anti-ABTS, and iron (III) reduction were 0.91, 0.86, and 0.99, respectively (*p* < 0.0001). Similar findings were shown in a previous study [16,29,30]. Additionally, it was found in this study that there was an insignificant correlation between the COX-2 and phenolic compound, contrary to a previous study [16].

## 4. Conclusions

This study highlighted the antioxidant, anti-inflammatory, and the phytochemical potential of six fractions from *B. engleri* and *B. erecta*, well-known medicinal plants of Burkina Faso. Their utilization could be supported partially by antiradical scavenging and iron (III) reduction. According to the interesting biological activity of *B. engleri*, the next step would be to isolate the anti-radical flavonoid from butanol, ethyl acetate, and acetonitrile fractions, as well as the anti-COX-2 and anti-LOX-15 compounds from the dichloromethane in vitro model.

## Figures and Tables

**Table 1:** Yield and antioxidant activity of fractions.

	Fractions	Yield * (mg/g)	DPPH (mgQE/g)	ABTS (mMTE/g)	FRAP (mgAAE/g)
*B. engleri*	Hexane fraction	15.94	Non-active	10.06 ± 1.33 ^ef^	10.20 ± 0.61 ^j^
	Dichloromethane fraction	12.11	2.25 ± 0.9 ^f^	8.67 ± 2.06 ^ef^	29.36 ± 0.75 ^hi^
	Acetonitrile fraction	19.17	17.88 ± 0.91 ^e^	11.42 ± 1.12 ^def^	64.34 ± 0.92 ^g^
	Ethyl acetate fraction	3.98	26.74 ± 1.84 ^d^	11.88 ± 0.91 ^cde^	74.61 ± 0.73 ^f^
	Methanol fraction	137.84	37.67 ± 0.88 ^c^	15.43 ± 1.04 ^bcd^	97.14 ± 3.55 ^e^
	Butanol fraction	1.23	51.88 ± 1.52 ^b^	17.10 ± 0.54 ^bcd^	211.68 ± 3.11 ^a^
*B. erecta*	Hexane fraction	13.87	Non-active	7.16 ± 1.40 ^f^	24.56 ± 1.54 ^i^
	Dichloromethane fraction	6.03	5.80 ± 0.18 ^f^	9.11 ± 0.96 ^ef^	37.02 ± 2.40 ^h^
	Acetonitrile fraction	20.79	64.14 ± 0.67 ^a^	22.86 ± 1.30 ^a^	174.16 ± 4.88 ^c^
	Ethyl acetate fraction	3.07	55.28 ± 3.46 ^b^	16.21 ± 1.75 ^bc^	126.65 ± 2.44 ^d^
	Methanol fraction	141.94	63.94 ± 0.78 ^a^	21.57 ± 1.82 ^a^	198.55 ± 4.54 ^b^
	Butanol fraction	1.67	41.40 ± 0.90 ^c^	14.86 ± 2.45 ^bcd^	92.02 ± 3.69 ^e^

Data in each column were statistically different letter (^a–j^) (*p* < 0.05) except data with same letters. The data were obtained in two independent triplate tests *(n* = 2 × 3). Aterisk (*) indicated data that were obtained by one procedure extraction. DPPH: 2,2-diphenyl-1-picrylhydrazyl, ABTS—2,2-*O*-azinobis(3-ethylbenzoline-6-sulphonate); FRAP—ferric (Fe III) reducing antioxidant power. mgQE/g: milligram quercetin equivalent per gram, mMTE/g: millimole Trolox equivalent per gram, mgAAE/g: milligram ascorbic acid equivalent per gram.

**Table 2:** Anti-inflammatory activities of fractions.

	Fractions	COX-2 (%Inhibition)	COX-1 (%Inhibition)	LOX-15 (%Inhibition)
*Bidens engleri*	Hexane fraction	42.74 ± 6.23 ^b^	Non active	Non active
	Dichloromethane fraction	64.72 ± 2.13 ^a^	Non active	62.55 ± 5.09 ^a^
	Acetonitrile fraction	31.17 ± 11.02 ^bc^	8.90 ± 1.15 ^c^	36.76 ± 3.59 ^b^
	Ethyl acetate fraction	37.39 ± 5.67 ^bc^	42.52 ± 0.90 ^a^	64.90 ± 4.78 ^a^
	Methanol fraction	32.31 ± 4.84 ^bc^	1.46 ± 0.46 ^d^	57.35 ± 0.01 ^a^
	Butanol fraction	37.87 ± 2.41 ^bc^	31.35 ± 2.30 ^b^	60.83 ± 0.80 ^a^
*Boerhavia erecta*	Hexane fraction	23.65 ± 2.55 ^c^	Non active	Non active
	Dichloromethane fraction	36.05 ± 2.19 ^bc^	Non active	Non active
	Acetonitrile fraction	31.10 ± 3.54 ^bc^	Non active	Non active
	Ethyl acetate fraction	26.44 ± 3.29 ^c^	Non active	Non active
	Methanol fraction	32.41 ± 3.08 ^bc^	Non active	Non active
	Butanol fraction	31.97 ± 6.07 ^bc^	Non active	Non active

Data in each column were statistically different letter (*p* < 0.05) except data with same letters (^a–j^). The data were obtained in triplate tests *(n* = 3).
